# Feasibility of conducting HIV prevention trials among key populations in Nairobi, Kenya

**DOI:** 10.1186/s12889-022-14875-2

**Published:** 2022-12-20

**Authors:** Elizabeth Mueni Mutisya, Vincent Muturi-Kioi, Andrew Abaasa, Delvin Nyasani, Rhoda W. Kabuti, Laura Lunani, Timothy Kotikot, Moses Mundia, Gaudensia Mutua, Geoffrey Ombati, Hannah Nduta, Matt A. Price, Joshua Kimani, Aggrey Omu Anzala

**Affiliations:** 1 KAVI-Institute of Clinical Research, University of Nairobi, Nairobi, Kenya; 2 IAVI, Nairobi, Kenya; 3 MRC/UVRI and LSHTM Uganda Research Unit, Entebbe, Uganda; 4 SWOP-PHDA, University of Nairobi/University of Manitoba, Nairobi, Kenya; 5 IAVI, New York, USA; 6 Department of Epidemiology and Biostatistics, University of California, San Francisco, California, USA; 7 School of Medicine, College of Health Sciences, University of Nairobi, Nairobi, Kenya

**Keywords:** Key population, Feasibility, HIV prevention, Clinical trials, Kenya

## Abstract

**Objective:**

To assess the feasibility of conducting HIV prevention trials among key populations in Nairobi, Kenya.

**Background:**

HIV prevention trials require the inclusion of those at high risk of HIV infection and their informed decision to take part and remain in the clinical trial to the end is crucial. In Kenya key populations including men who have sex with men (MSM) and female sex workers (FSW) are, disproportionately, at high risk of HIV infection when compared to the general population. Few trials testing biomedical prevention products against HIV have enrolled Kenyan FSW and MSM.

**Methods:**

We performed simulated vaccine efficacy trial (SiVET) using licensed hepatitis B vaccines as substitutes for a HIV vaccine candidate and included randomization for those immune to hep B. The SiVET was an observational study designed to mimic the rigors of a clinical trial; we assessed HIV risk, provided risk counselling and prevention tools and performed HIV testing at baseline and periodically until the end of the trial. MSM and FSW were enrolled at a ratio of 4:1. Volunteers were assigned to either hepatitis B vaccine or placebo.

**Results:**

Recruitment took approximately 24 months between Sep 2015 and Sep 2017. Of the 368 volunteers screened, 250 (200 MSM and 50 FSW) were enrolled. Reasons for exclusion at screening included: being positive for HIV (*n* = 7), hepatitis (*n* = 14), other pre-existing medical conditions (*n* = 41), eligible but chose not to enrol (*n* = 47). Most of the volunteers adhered to study procedures and attended their study visits within the study window. These include volunteers who received the second vaccination 244 (98%), the third vaccination 228 (91%) and, the final study visit 217 (87%). The reasons volunteers discontinued from the study early included: relocation and loss to follow up (*n* = 14). A total of 8 cases of HIV infection were observed in 174.5 Person Years at Risk (PYAR), all among MSM, including 5 seroconversions identified at the last study visit, for a HIV incidence of 4.58 cases/ 100 PYAR, among MSM enrolled in the study.

**Conclusion:**

Our findings suggest that it is possible to conduct HIV prevention trials among key populations in Nairobi with a good adherence to a vaccine efficacy trial schedule. Despite HIV prevention efforts, we also noted a high incidence of HIV infection. This demonstrates the need for effective HIV prevention products in these populations.

## Background

The significance of any clinical trial is dependent on the quality of data [1, 2]. The randomized clinical trial provides the highest level of evidence on the efficacy and safety of biomedical products and is almost always required prior to the licensure of new products [3–5]. Effective volunteer recruitment, enrolment, adherence to protocol schedules and procedures and retention within the trial are key elements for a successful clinical trial [2, 6–9].

Prevention of HIV infection is key to ending the pandemic [10, 11]. HIV prevention trials will require the inclusion of those at high risk of HIV infection [1, 12, 13] and their decision to take part and remain in the clinical trial to the end is important [1, 14, 15]. Globally men who have sex with men (MSM) and female sex workers (FSW) are at a disproportionately high risk of HIV infection with a higher HIV incidence and prevalence compared to the general population [10, 11]. In Kenya, MSM HIV incidence is estimated to be at 10.9 per 100 person-years of follow up [16], and estimates for FSW stand at an annual incidence of 2.2% [17]. In Nairobi, the number of MSM and FSW at high risk of HIV infection is estimated to be 11,042 and 39,494 respectively [18, 19]. The population of those at high risk of HIV infection and the high HIV incidence in these groups means they would be the primary beneficiaries of new biomedical prevention products against HIV. This high HIV incidence makes these groups attractive to approach when considering populations for future HIV prevention trials; however, few trials have enrolled Kenyan MSM and FSW.

Studies assessing the feasibility of enrolling and retaining high risk populations into HIV prevention trials provide confidence to those intending to conduct trials in these communities, and provide a case for the inclusion of those most likely to benefit from the licensure of these products [1, 20, 21]. Such studies ensure a realistic evaluation of the suitability of these populations to participate in clinical trials [2, 22, 23]. Simulated studies allow researchers to gather information on risks of HIV infection in the context of a trial environment, extent of HIV risk behaviour and assess whether or not high risk volunteers can be enrolled and retained in a clinical trial setting while adhering to study visits and procedures [2, 14, 21, 24].

Simulated vaccine efficacy trials (SiVETs) have been designed to determine the suitability of the target population and inform recruitment rates, adherence with protocol procedures and retention of volunteers in a trial that mirrors the rigors of an actual HIV prevention trial. SiVETs have been used to identify population- specific challenges prior to the start of an efficacy trial, and provide data to inform clinical operations decisions. These studies have been conducted elsewhere and have yielded reliable results [7, 21, 23–25]. In Uganda, SiVET studies have been used to assess willingness to participate in future HIV vaccine trials among MSM, FSW and fisher folk, as well as the feasibility of carrying out these trials in these populations [21, 26–28].

Between 2015 and 2018, we performed a placebo-controlled, randomized, SiVET among MSM and FSW in Nairobi, Kenya where hepatitis B vaccines were used as a substitute for an actual HIV vaccine candidate. The volunteers completed between 12 and 15 months of follow-up in the study designed to mirror an actual HIV prevention trial to determine the feasibility of conducting future HIV prevention trials among key populations living in Nairobi, Kenya. We have previously reported results from this study, demonstrating a high willingness to participate in HIV vaccine trials among members of this population. This was associated with study volunteers reporting having had a good experience in the study [14]. Here, we present results from the evaluation of recruitment rates, retention rates, adherence to study procedures and volunteer suitability for future HIV biomedical clinical trials.

## Methods

### Design and setting

As previously outlined [14], community engagement, demand creation and recruitment activities were conducted by the Sex Workers Outreach Program (SWOP) clinics serving FSW and MSM in Nairobi. SWOP- clinics offer services in a FSW and MSM friendly setting including: HIV counselling and testing, HIV risk reduction counselling, diagnosis, treatment and care for sexually transmitted infections (STI), anti-retroviral treatment (ART), Pre-Exposure Prophylaxis (PrEP) against HIV, Post-Exposure Prophylaxis (PEP) against HIV, condoms and family planning services. SWOP serves a population of approximately 24,500 active and self-reported sex workers, both men and women. Potential volunteers were referred to the KAVI- Institute of Clinical Research (KAVI-ICR) at the University Of Nairobi, Kenya. KAVI-ICR has two sites – one hospital based at the Kenyatta National Hospital and one community based at the Kangemi region on the western part of Nairobi.

After informed consent, participants were screened for eligibility and this included testing for HIV using two rapid antibody test kits carried out in parallel in line with national recommendations at the time. A third rapid test was used for samples with discordant results and confirmatory testing was done for samples with positive rapid tests using a Bioelisa HIV1 + 2 Ag/Ab test kit.

HIV negative volunteers were tested for hepatitis B surface antigen (HBsAg), antibodies to hepatitis B surface antigen (anti-HBs), and antibodies to hepatitis B core antigen (anti-HBc) using bioelisa HBsAg, anti-HBs and anti-HBc respectively. Volunteers identified to be susceptible to hepatitis B infection (anti-HBs negative) were all assigned to receive the hepatitis B vaccine. Volunteers who did not have an ongoing hepatitis B infection, and were identified to be immune to hepatitis B either due to a previous infection or vaccination, were randomized to receive either the vaccine or placebo (1:1). Study volunteers and site staff (except the pharmacist and the laboratory staff) remained blinded until the end of the study.

Volunteers who were hepatitis B uninfected with no prior immunization or exposure were provided with 3 doses of a hepatitis B vaccine at baseline, 1 month and 6 months. Those who were positive for antibodies to the hepatitis B surface antigen (anti-HBs), either due to previous infection or vaccination, were randomly assigned to receive either placebo or the hepatitis B vaccine at baseline, 1 month and 6 months. Initial volunteers were assigned to be followed for 15 months; this was later amended to 12 months due to funding considerations.

### Sample size

A pre-determined sample size of 250 for the SiVET was calculated based on being able to estimate one-year retention of 80% with a precision of ±5, 80% power and two-sided level of significance of 5%. The target was to enrol 200 MSM and 50 FSW. We deliberately chose to enrol 20% FSW in the interest of preventing stigma against one group and avoiding any negative feelings among the SWOP-clinics clients [14].

### Recruitment

Recruitment was organized at SWOP clinic staff and affiliated peer outreach workers. They identified potential volunteers from the clinics and hot spots within the community. Those interested were provided with appropriate information about the SiVET study. Hot spots are areas such as streets corners, clubs, lounging, bars, hotels, massage parlours in Nairobi where sex workers meet their clients. Community sensitization about the research was carried out by trained FSW peer sex workers, MSM peer educators, prevention officers and the SWOP clinical team.

Members of the community who showed interest in participation were referred to a central SWOP clinic to be pre-screened by trained clinic staff. They were provided with general information on study requirements, procedures and duration, the inclusion and exclusion criteria and samples to be collected at KAVI-ICR site. HIV-uninfected MSM and FSW who were 18 and older, residing in Nairobi and registered in SWOP for at least 3 months were referred by the SWOP peer outreach workers to KAVI-ICR.

At KAVI-ICR, study education sessions were led by nurse counsellors. Depending on the individual level of understanding, each volunteer had up to three sessions of detailed discussions about the study and had all their questions addressed before they signed the informed consent and were screened for eligibility.

### Screening and vaccination

Screening included rechecking the inclusion criteria of age requirements, Nairobi residence and follow up at SWOP clinics, as well as being, sexually active in the preceding 3 months. Volunteers also had to be willing to: undergo HIV risk assessment and HIV testing, to provide contact information and to be contacted by study staff, and to return for study visits.

Volunteers were excluded if: HIV infected, had ongoing hepatitis B infection, known to be pregnant or nursing mothers, not available to come to the clinic regularly for follow-up, had prior severe reactions to vaccines or had any significant clinical condition as assessed by the investigator.

Female volunteers were educated on effective family planning methods; long-acting reversible contraceptives, including injectables, implants and intra-uterine devices, were deemed appropriate and in keeping with what would be expected in a clinical trial. Injectable contraceptives were provided at study site while those who required other methods were referred to a family planning clinic. The contraceptive method of choice and compliance was documented and confirmed at designated study visits.

Eligible volunteers that were negative for hepatitis B antibodies and antigens at screening were assigned to receive a hepatitis B vaccine (ENGERIX-B™ GlaxoSmithKline Biologicals Rixensart, Belgium or EUVAX-B Sanofi Pasteur ltd, Korea) at baseline, 1 month and 6 months. Volunteers with evidence of immunity to hepatitis B were assigned randomly to either receive a hepatitis B vaccine or placebo; volunteers and investigators were blinded to study arm. At month 9 of the trial those who were found to have anti-HBs serum titres of ≤10 IU/L were counselled and offered revaccination.

### Follow up and volunteer retention

Volunteers were asked to return for follow-up study visits at months 3, 9 and 12 or 15 post enrolment (Table 1.). During these visits, assessment for any adverse events, HIV testing and risk assessment, sexually transmitted infection testing, contraceptive counselling and pregnancy testing were performed. Following each vaccination visit (enrolment, months 1 and 6), volunteers were asked to return in 7 days to assess adverse events after vaccination.

**Table 1.** Study visit schedule for volunteers enrolled in a SiVET study in Nairobi, Kenya, between September 2015 and September 2018

Schedule of study visits											
Study visit	Screening	2	2B	3	3B	4	5	5B	6	7	8
Study Month (M = 28 days)		M0		M1		M3	M6		M9	M12	M15
Visit Window (Days)	−28		±2	±3	±2	±3	±3	±2	±3	±3	±3
Vaccination visit		X		X			X				
7 day- post vaccination			X		X			X			
Follow up visit						X			X	X	X

Each volunteer was provided with an individualised study visit calendar. Free treatment for common illnesses was provided as needed at the study clinic and those who required specialised treatment were referred appropriately. The study site covered the cost of laboratory investigations and specialised treatment provided elsewhere. Mobile phone communication cost that would be required to make urgent calls at vaccination visits was provided. Study nurses informed volunteers of their next scheduled date at each study visit. Locator information was updated at each visit and a phone call reminder to the volunteers was performed a day before their actual scheduled clinic visit. Those who missed a visit were traced and followed up by a field liaison officer to encourage them to come to the clinic. Up to three attempts were made to reach the volunteer using the contact and locator information provided.

### HIV risk assessment, HIV testing and counselling and referral for care

At enrolment, we administered a questionnaire on socio-demographics and HIV risk behaviour. Socio-demographics included volunteers being asked about their age, level of highest education. Assessment of HIV risk behaviour comprised volunteers being asked; how often they had a drink containing alcohol, number of sex partners, new sex partners, the HIV status of sex partners, use of any illicit drugs, if they had insertive or receptive anal sex, and how frequently they used condoms when having sex in the preceding month. An alcohol use disorder screening tool, the CAGE questionnaire [29], was administered. CAGE has four questions and the responses for each are assigned a score of 0 or 1; a score greater than 2 is an indication of heavy alcohol use or an alcohol use disorder. We evaluated HIV prevention knowledge to assess both biomedical and behavioural prevention methods at the last study visit [14].

At baseline, months 1, 3, 6, 9, and at the last study visit, trained study nurses performed HIV pre-test counselling prior to collecting a finger prick of blood for an HIV parallel test (Alere determine HIV1/2™ (Alere Medical Co Ltd., Matsuhidai, Matsudo-shi, Chiba, Japan) and Unigold™ Recombigen® HIV ½ (Trinity biotech PLC Bray,co.Wicklow, Ireland place), and HIV post- test counselling after the results were available, according to the Kenya national guidelines [30]. Those who were found to be HIV infected were counselled and provided with coping strategies and referred for care and treatment. HIV uninfected volunteers were counselled on risk reduction based on reported individual risk to prevent future HIV infection. Early diagnosis and treatment of other sexually transmitted infections and referral for voluntary medical male circumcision was provided. Condoms, PEP and water-based lubricants were offered to all volunteers as needed at study visits.

### Study outcomes

Study outcomes included: (a) Speed of study enrolment, (b) Volunteer retention and adherence to study schedule i.e. attending all study visits within visit window (Table 1.), and (c) HIV incidence.

Study completion was defined as completing all study visits or up to seroconversion visit for volunteers that became HIV infected.

### Statistical methods

Data were captured and managed in OpenClinica version 3.0 and analysed in STATA version 14.0. We summarised sociodemographic, HIV risk behaviour at baseline using counts and percentages overall and stratified by population (MSM or FSW). We estimated the proportion of volunteers that received all the three vaccinations as number completing all vaccinations divided by the total number of enrolled volunteers. We estimated adherence to study procedures as those who had attended all their visits within the study window divided by total number of volunteers studied, expressed as a percentage. Retention was estimated as number of volunteers that completed the study as per the study completion definition above, divided by the total number of volunteers enrolled. We fitted logit models for both univariate and multivariable analysis to determine factors associated with retention. Factors that were associated with retention at univariate analysis *p* < 0.20 on log likelihood ratio test were considered for multivariable analysis, except for sex and drug use (based on previous studies) which were included a priori. Factors were retained in the multivariable logit model if the log likelihood ratio test *p*-value of inclusion of a factor was ≤0.05. HIV incidence was estimated as total number of HIV positive cases divided by total number of person years at risk (PYAR) stratified by gender, expressed as per 100 PYAR. Person-years at risk were calculated as the sum of the time from baseline to the date of the last HIV-uninfected result, or to the estimated date of HIV infection for each volunteer. Date of HIV infection was imputed as the mid-point of the interval between the last HIV-uninfected and the first HIV-infected result dates.

## Results

### Recruitment, screening and enrolment of study population

**Fig. 1:**
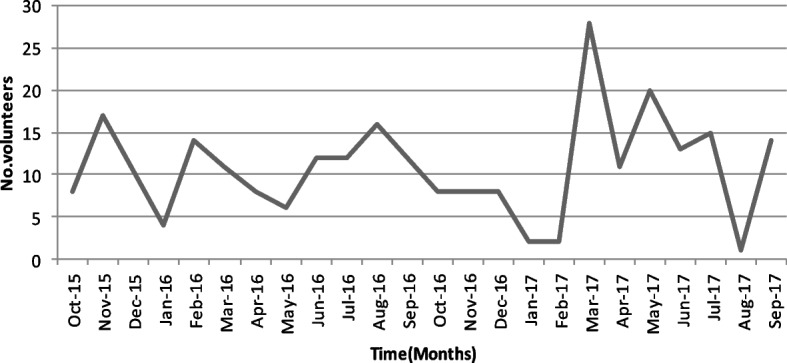
Enrollment rate among volunteers enrolled in the SiVET study in Nairobi, Kenya between 2015 and 2017

Recruitment took approximately 24 months between Oct 2015 and Sep 2017 when all volunteers were enrolled (Fig. 1).

**Fig. 2:**
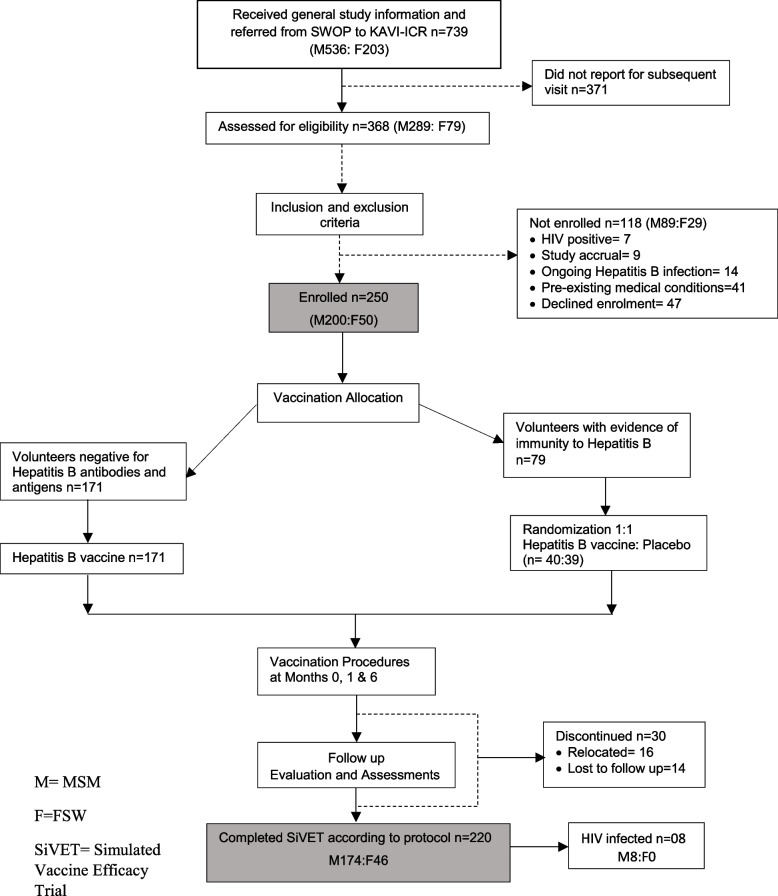
Flow diagram of volunteer recruitment, screening, enrolment, vaccination, and retention in a SiVET study among MSM and FSW in Nairobi, Kenya September 2015–September 2018

Total 739 potential volunteers identified in the community were referred to the KAVI-ICR study clinic for screening (Fig. 2). Of these, 371(50.2%) did not report for subsequent enrolment visit; 368 (49.8%) were screened for enrolment in the SiVET and 250 (68%) met the eligibility criteria and were enrolled. The main reasons for exclusion at screening were: being HIV positive (*n* = 7), ongoing hepatitis B infection (HBsAg+) (*n* = 14), other pre-existing medical conditions (*n* = 41), eligible volunteers choosing not to enrol (*n* = 47) and study accrual with the study site having met the enrolment target (*n* = 9).

At enrolment, 171 volunteers tested negative for hepatitis B antibodies and the surface antigen and received hepatitis B vaccine while 79 who tested positive for hepatitis B antibodies were randomized to vaccine or placebo (1:1). Of those enrolled, 211 received hepatitis B vaccine and 39 were randomized to receive placebo.

### Volunteer characteristics and trial retention

Volunteer characteristics are presented in Table 2. The study population was made up of 200 MSM and 50 FSW. The majority of the male volunteers were aged 18–24 years while most of the women enrolled in the study were older than 25 years of age. Over 80% of volunteers had secondary or tertiary education. Several volunteers reported engaging in risky sexual behavior with nearly 39% reporting alcohol use before sex and more than 49% having 3 or more sexual partners in the month preceding their participation in the study. The majority of the volunteers demonstrated knowledge for behavioral and biomedical methods of HIV prevention and 80% reported always using condoms during sexual intercourse in the month prior to their screening visit.

**Table 2:** Characteristics of volunteers enrolled and retained in a SiVET study, stratified by MSM and FSW status, in Nairobi, Kenya between September 2015 and September 2018

Characteristic	All Enrolled		Men who have sex with men (MSM)				Female sex workers (FSW)			
	***N*** = 250		Enrolled ***N*** = 200		Completed study ***N*** = 174		Enrolled ***N*** = 50		Completed study ***N*** = 46	
	N	(%)	N	(%)	N	(%)	N	(%)	N	(%)
Total	**250**	**(100)**	**200**	**(80)**	**174**	**(87)**	**50**	**(20)**	**46**	**(92)**
Age, years										
18–24	113	(45)	105	(52)	84	(48)	8	[16]	8	[17]
25–34	82	[33]	61	[31]	57	[33]	21	[42]	18	[39]
≥ 35	55	[22]	34	[17]	33	[19]	21	[42]	20	[44]
Highest level of education Completed										
Primary	48	[19]	24	[12]	21	[12]	24	(48)	21	(46)
Secondary	132	(53)	111	(55)	97	(56)	21	[42]	21	(45)
vTertiary/Higher	70	[28]	65	[33]	56	[32]	5	[10]	4	(09)
Alcohol use in the last month										
Never	89	[36]	74	[37]	64	[37]	15	[30]	14	[30]
Sometimes	161	(64)	126	(63)	110	(63)	35	(70)	32	(70)
Alcohol before sex in the last month										
Never Sometimes	153 94	(61) [38]	127 70	(63) [35]	11,062	(63) [36]	26 24	(52) (48)	23 23	(50) (50)
Always	3	(01)	3	(02)	2	(01)	0	(00)	0	(00)
Alcohol CAGE score										
≤ 1	220	(88)	177	(88)	155	(89)	43	(86)	40	(87)
≥ 2	30	[12]	23	[12]	19	[11]	7	[14]	6	[13]
Illicit drug use in the last month										
No	186	(74)	141	(70)	125	(72)	45	(90)	42	(91)
Yes	64	[26]	59	[30]	49	[28]	5	[10]	4	(09)
Number of sex partners in the last month										
1	71	[28]	62	[31]	53	[31]	9	[18]	7	[15]
2	56	[23]	50	[25]	44	[25]	6	[12]	6	[13]
≥ 3	123	(49)	88	[44]	77	[44]	35	(70)	33	(72)
New sex partners in the last month										
No	124	(50)	114	(57)	98	(58)	9	[20]	9	[20]
Yes	126	(50)	86	[43]	72	[42]	37	(80)	37	(80)
Insertive Anal sex in the last month										
No	50	[25]	50	[25]	44	[25]	N/A	N/A	N/A	N/A
Yes	150	(75)	150	(75)	130	(75)	N/A	N/A	N/A	N/A
Receptive anal sex in the last month										
No	157	(63)	107	(54)	90	(52)	50	(100)	46	(100)
Yes	93	[37]	93	(46)	84	(48)	0	(00)	0	(00)
Sex with HIV infected partners										
No	70	(28.0)	66	(33.0)	60	[34]	4	(08)	3	(07)
Yes	4	(1.6)	3	(1.5)	3	(02)	1	(02)	1	(02)
Don’t know	176	(70.4)	131	(65.5)	111	(64)	45	(90)	42	(91)
Condom use in the last month										
Sometimes	51	[20]	47	[24]	42	[24]	4	(08)	3	(07)
Always	199	(80)	153	(76)	132	(76)	46	(92)	43	(93)
HIV behavioral preventive knowledge										
Yes	250	(100)	200	(100)	171	(100)	50	(100)	46	(100)
No	0	(00)	0	(00)	0	(00)	0	(00)	0	(00)
HIV biomedical preventive knowledge										
Yes	155	(62)	116	(58)	99	(57)	39	(78)	36	(78)
No	95	[38]	84	[42]	75	[43]	11	[22]	10	[22]

*N/A* Not applicable

Volunteers were initially invited to 15 months of follow up, but due to financial constraints we reduced the follow up to 12 months with a total of 127 volunteers followed up to 15 months. Of the 250 total enrolled, 220 (88%) volunteers completed the trial follow-up according to protocol or reached a study endpoint; these included three volunteers who became HIV infected before the last scheduled study visit and underwent an early termination visit as required in the protocol. A total of 217(87%) volunteers attended the last study visit. Those retained in the trial included 174 MSM (87%) and 46 FSW (92%). Nearly half of the volunteers who did not complete the study reported relocating outside of study area 16 (53%), the remainder 14(47%) were lost to follow up (unreachable/uncontactable).

### Predictors of trial retention

Table 3 shows predictors of trial retention in future HIV prevention trials in univariate analyses and in a final multivariable model. In the adjusted analysis, only age remained a significant predictor of retention, with being above 35 years of age (aOR 8.19, 95% CI 1.72–38.93, *P*-value 0.008) and between 25 and 34 years of age (aOR 3.12, 95% CI1.16–8.40, P-value 0.025) was independently associated with increased retention in the trial when compared to those between 18 and 24 years of age.

**Table 3:** Predictors of retention in future HIV preventive trials among volunteers enrolled in a Simulated Vaccine Efficacy Trial in Nairobi, Kenya: Between September 2015 and September 2018

Predictor	Retention				LRT-			
	N	(%)	uOR	(95%CI)	p-value	aOR	(95%CI)	p-value
Overall retention	220	(88)	**–**		**–**		**–**	**–**
Gender					0.310			
Male	174	(87)	1.00			1.00		
Female	46	(92)	1.72	(0.57–5.17)		0.86	(0.23–3.18)	0.827
Age, years					0.007			
18–24	92	(81)	1.00			1.00		
25–34	75	(92)	2.45	(1.00–6.06)		3.12	(1.16–8.40)	0.025
≥ 35	53	(96)	6.05	(1.36–26.82)		8.19	(1.72–38.93)	0.008
Highest level of education Completed					0.744			
Primary	42	(88)	1.00					
Secondary	118	(89)	1.20	(0.43–3.34)				
Tertiary/Higher	60	(86)	0.86	(0.29–2.546)				
Alcohol use in the last month					0.897			
Never	78	(88)	1.00					
Sometimes	142	(88)	0.86	(0.29–2.33)				
Alcohol before sex in the last month					0.438			
Never	133	(87)	1.00					
Sometimes	85	(90)	1.42	(0.62–3.27)				
Always	2	(67)	0.30	(0.03–3.47)				
Alcohol CAGE score					0.421			
≤ 1	195	(89)	1.00					
≥ 2	25	(83)	0.64	(0.23–1.83)				
Illicit drug use in the last month					0.152			
No	167	(89)	1.00			1.00		
Yes	53	(83)	0.55	(0.25–1.23)		0.48	(0.20–1.18)	0.089
Number of sex partners in the last month					0.578			
≤ 1	60	(85)	1.00					
2	50	(89)	1.53	(0.53–4.42)				
≥ 3	110	(89)	1.55	(0.65–3.67)				
New sex partners in the last month					0.963			
No	109	(88)	1.00					
Yes	111	(88)	1.02	(0.47–2.18)				
Receptive anal sex in the last month					0.378			
No	136	(87)	1.00					
Yes	84	(90)	1.44	(0.63–3.29)				
Condom use in the last month					0.953			
Sometimes	45	(88)	1.00					
Always	175	(88)	0.97	(0.37–2.52)				
HIV biomedical preventive knowledge					0.572			
Yes	135	(87)	1.00					
No	85	(90)	1.79	(0.35–1.78)				

### Adherence to study procedures

The majority of the volunteers adhered to study procedures and attended their study visits within the study window (Table 4). This included volunteers who attended the second vaccination 229(92%), third vaccination 208(83%) and last study visit, 179(72%). The number of visits outside of the study window or missed study visits increased with longer duration between visits and the most, 38 visits outside of the study window and 33 missed study visits occurred between month 9 and last study visit when the visit interval was longest, between 3 and 6 months.

**Table 4:** Trial visit attendance of the 250 volunteers enrolled in SiVET by timelines and events September 2015–September 2018

Timelines and events																
Scheduled study visits	Vaccination and Follow up visits N = 250															
Study Visit Attendance(m = months)	M0^*^ N (%)		M1^*^ N (%)		M3 N (%)		M6^*^ N (%)		M9 N (%)		M12 N (%)		M15 N (%)		M12/15 N (%)	
Visits within study window	250	(100)	229	(92)	207	(83)	208	(83)	193	(77)	93	(76)	86	(68)	179	(72)
Visits Outside study window	n/a		15	[6]	30	[12]	20	[8]	32	[13]	12	[10]	26	[20]	38	[15]
Missed study visits	n/a		6	[2]	13	[5]	22	[9]	25	[10]	17	[14]	16	[12]	33	[13]

n/a = not applicable.

* = Vaccination visit

### HIV incidence

At the end of the study period, a total of 8 male cases of HIV infection were observed in 174.5 male PYAR, including 5 seroconversions identified at the last study visit, translating into a HIV incidence among MSM of 4.58 cases/ 100 person years of follow up, 95% CI: 2.29–9.17. No cases of incident HIV were detected in 53 PYAR among FSW.

## Discussion

Our results show that, while challenging, it is feasible to recruit, follow-up and retain key populations at high risk of HIV infection in Nairobi. Among MSM and FSW from Nairobi who were recruited, about nine of ten were retained and completed the trial. Our findings suggest that future HIV prevention trials among key populations are possible with good adherence to a vaccine efficacy trial schedule.

Our study found age to be significantly associated with retention. Younger volunteers between 18 and 24 years were less likely to remain in the study to the end compared to volunteers above 25 years. Similar findings were reported in other studies among high risk women in fishing communities in Uganda and Kenya where drop out was more likely among those below 35 years [25, 31] and among young gay men in USA [32]. However, to demonstrate efficacy of new HIV prevention products, a process which is dependent on HIV incidence and duration of trial follow-up [1, 9, 24], there is a need to balance between enrolling volunteers at the highest risk of infection (which can be associated with youth) and those who are most likely to be retained in the trial.

The duration of recruitment was longer than anticipated because of a slow initial rate of enrolment. Over 40% of the volunteers were enrolled in the last 6 months. Initially we did experience challenges identifying HIV negative volunteers who were willing to reveal their sexual orientation and participation in sex work. FSW and MSM are hard-to-reach volunteers that are highly mobile, and we faced problems accessing and working in challenging environments including areas in purportedly dangerous neighbourhoods, bars, and informal housing, all of which provided challenges in identifying potential volunteers. Early feedback from volunteers suggested a discomfort in moving from their familiar SWOP clinic to the study clinic. Study clinic staff received additional sensitivity training. To increase confidence in attending study clinic visits outside the SWOP, we adopted a strategy to contact/trace volunteers in their locality. Staff training at the study clinics to offer a friendlier environment was part of this strategy.

However, we found that retention rates exceeded our estimated retention rate of 80%, contrary to the perception that key populations may be difficult to retain in clinical trials [33] . We demonstrated a high retention rate and minimal trial dropout among the high risk groups comparable to other studies done elsewhere [2, 27, 32, 34] and higher than studies done in Uganda [35, 36]. High dropout rates in a clinical trial undermine the validity of study findings as individuals who drop out may differ from those who complete trials [37].

We attribute the high retention to the recruiting and retention strategies used, including the sensitivity training of staff. SWOP clinics offer FSW and MSM friendly services and the study clinic staff had trained on how to interact with key populations. Both study teams in SWOP clinics & KAVI-ICR communicated regularly on retention issues. During these meetings, they examined the recruitment approaches, follow up and management of hard –to- reach volunteers and came up with ideas on how to improve volunteer experiences during study participation. Both SWOP and KAVI-ICR adopted a dynamic approach to community engagement and volunteer recruitment.

Among those who did not complete the trial, loss to follow-up and relocation from trial area was the main reasons given. Our findings are comparable to those from a study in Uganda among a high risk population of fisher-folk [35, 36] and non-fisher folk [38]. Of those who relocated, some migrated to other towns within the country while others relocated to other countries. Among those who relocated outside of the country were MSM of Ugandan origin that had sought asylum in Kenya. Those who were granted asylum outside of Kenya left the country without notice and informed the study team once they settled in their new locations. Migration has been associated with an increased risk of HIV infection, due to reduced access to HIV prevention modalities [39] coupled with increased high risk sexual behaviour among migrants [38, 40] . These findings reinforce the need to consider strategies and trial designs that would provide an opportunity for inclusion of migrant communities in HIV prevention trials in order to address the unmet medical need for those at high risk of HIV infection [41, 42].

Maximizing adherence to trial protocols will be imperative in HIV preventive trials [2, 22, 23]. Our study shows a good adherence to procedures with majority of the visits occurring within the study window. One reason for the high schedule adherence among volunteers might be the strong relationships they have with their care providers at SWOP clinics. It is also possible that the volunteers who chose to participate in this study were more adherent to their visits than the overall population at these clinics. However, we observed a small proportion of missed study visits and visits occurring outside of the study window. Including more flexible visit windows as part of clinical trials design could result in a greater opportunity for volunteers to adhere to study visits.

Despite the availability of a high standard of prevention for all volunteers, we noted a high incidence of HIV infection among MSM comparable to other studies done elsewhere [13, 21, 27, 32, 43].This incidence demonstrates an unmet need in these populations and would allow for measurement of the efficacy of HIV prevention products. Incidence rates are liable to change over the course of study and may be affected by the rate at which volunteers are lost to follow-up and HIV prevention interventions available to study volunteers [7, 44].

The strengths in this trial included a reasonable MSM sample size of an often hard-to-reach population that is reflective of the members of the community at high risk of HIV infection. However, the study had limitations; the sample size of 50 FSW would make it difficult to accurately compare the group’s outcomes with those in the MSM group.

The relatively small size of the study, with few outcomes (33 drop outs) meant our statistical power to detect correlates of retention was not ideal; we only observed that age was a significant predictor. The SiVET used licensed commercially available hepatitis B vaccines in place of an experimental product; hence observed outcomes might not fully represent a trial using a real HIV prevention investigational product with an unknown safety profile.

Based on the challenges with recruitment that saw a significant proportion of participants that were referred for screening decline to participate, the study population may not be representative of the general population of MSM and FSW at risk of HIV in Nairobi. We did not collect any details on participants during the referral process, so we are not able to compare those who came in for screening to those who declined the referral. Although this study has demonstrated the feasibility of successfully recruiting and retaining this population in a HIV vaccine efficacy trial, the trial population may not be suitable for assessment of acceptability of these new interventions in these populations.

The study was concluded in 2018, since then, new products and strategies, including oral PrEP and treatment as prevention have become widely available in Kenya. In addition, the COVID-19 pandemic has increased awareness globally on the process of vaccine development. Although our study provides valuable data to inform the conduct of efficacy trials with key populations in Nairobi, we acknowledge that these factors could affect the feasibility of conducting HIV prevention efficacy trials in key populations in Nairobi.

The experience of a study designed to mirror the rigors of an efficacy trial may provide some insight regarding actual clinical trial participation, hence may serve as a more valuable gauge of feasibility compared to hypothetical scenarios where volunteers are simply informed about clinical trials.

## Conclusion

We have previously reported results from this study demonstrating a high willingness to participate in HIV vaccine trials among members of these key populations from Nairobi, Kenya [14]. Here, we report a high level of retention among study volunteers. Our findings suggest that it is possible to conduct HIV prevention trials among key populations in Nairobi with good adherence to a vaccine efficacy trial schedule. Despite HIV prevention efforts, we also noted a high incidence of HIV infection. This demonstrates the ongoing need for more effective HIV prevention products for these populations.

## Data Availability

The datasets used and analysed during the study are available from URL https://doi.org/10.3886/E119622V1.

## Abbreviations

ART: Ant-retroviral treatment
FSW: female sex workers
ICR: Institute of clinical research
MRC/UVR and LSHTM: Medical Research Council/Uganda Virus Research Institute and London School of Hygiene and Tropical Medicine
MSM: Men who have sex with men
PHDA: partners for health and development for Africa
PEP: Post-Exposure Prophylaxis against HIV
PrEP: Pre-Exposure Prophylaxis against HIV
PYAR: Person Years at Risk
SiVET: Simulated vaccine efficacy trials
STI: Sexually transmitted infections
SWOP: Sex workers outreach program
USA: United states of America
USAID: United states agency international development
